# Supplementary material for: A microscopic model of wave-function dephasing and decoherence in the double-slit experiment
Source: Sci Rep. 2021 Oct 25;11:20986. doi: 10.1038/s41598-021-99995-2 (PMC8545942; doi:10.1038/s41598-021-99995-2)

APPENDIX I

Consider the geometry shown in Fig. 14.

FIG. 1. Geometry of the Double Slit Experiment

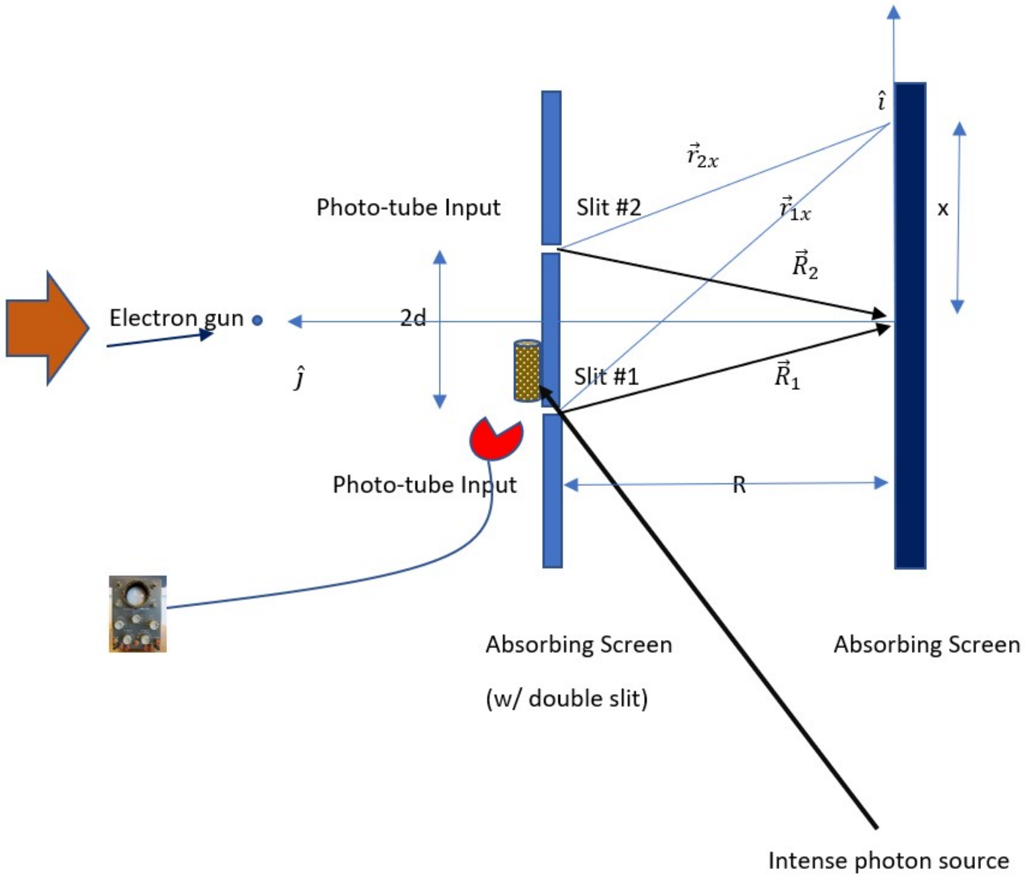

$$\vec{R}_1 = +d\,\hat{\imath} - R\,\hat{\jmath}$$
$$\vec{R}_2 = -d\,\hat{\imath} - R\,\hat{\jmath}$$

$$\vec{r}_{1x} = (x+d)\hat{\imath} - R\,\hat{\jmath} = \vec{R}_1 + x\,\hat{\imath}$$
$$\vec{r}_{2x} = (x-d)\hat{\imath} - R\,\hat{\jmath} = \vec{R}_2 + x\,\hat{\imath}$$

We can compute the wave-function at the point x above the mid-point between the slits. We can think of the slits as the sources of spherical waves and write the wave-function at x as the sum of spherical waves from each slit, *i.e.,*

$$\psi_1(x) = \frac{e^{ikr_{1x}}}{\sqrt{4\pi}\,r_{1x}^2}$$
$$\psi_2(x) = \frac{e^{ikr_{2x}}}{\sqrt{4\pi}\,r_{2x}^2}$$
$$\psi(x) = \frac{\psi_1(x) + \psi_2(x)}{\sqrt{2}} \quad (1)$$

where r_{1x}, r_{2x} are as defined in Fig. 14. The k is defined from the electron's momentum $\hbar k$. Note that we have two

interesting limits, *i.e.,* $d \ll R \sim x$ and $d \ll R \ll x$. We write (see behavior in Fig. 15)

$$r_{1x} - r_{2x} \equiv \Phi = \begin{cases} \frac{2dx}{R} & \text{if } d \ll R \sim x \\ 2d & \text{if } d \ll R \ll x \end{cases} \tag{2}$$

With this, we compute

$$P(x) = |\psi(x)|^2 = \frac{2}{(R^2+d^2)^2} \frac{1}{(1+\frac{x^2}{R^2+d^2})^2 - \frac{4d^2x^2}{(R^2+d^2)^2}} \left[\frac{(1+\frac{x^2}{R^2+d^2})^2 + \frac{4d^2x^2}{(R^2+d^2)^2}}{(1+\frac{x^2}{R^2+d^2})^2 - \frac{4d^2x^2}{(R^2+d^2)^2}} + \cos\Phi \right] \tag{3}$$

FIG. 2. Φ *vs.* x for $d = 1, R = 10$

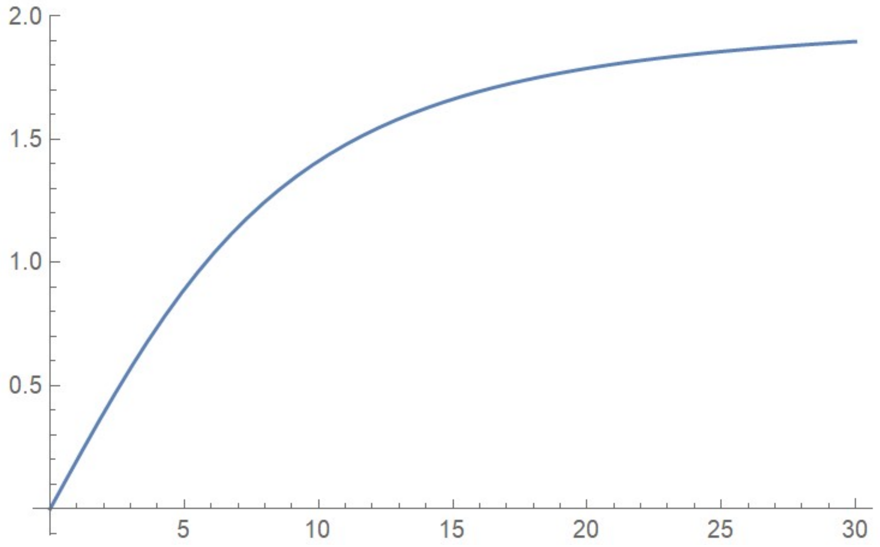

We have the limiting forms

$$P(x) = \begin{cases} \frac{4\cos^2\frac{\Phi}{2}}{R^4} & \text{if } d \ll R \sim x \\ \frac{4\cos^2\frac{\Phi}{2}}{x^4} & \text{if } d \ll R \ll x \end{cases} \tag{4}$$

which we plot (as an example for, additionally $k = 20\pi$)

APPENDIX II

A matrix $\mathcal{G}$ is positive semi-definite if $x^T \mathcal{G} x \geq 0$ for any vector x. Consider the matrix defined as

$$\mathcal{G} = \begin{pmatrix} a_1^2 & 0 & a_1a_2 & 0 & 0 & \dots & \dots & \dots & \dots & 0 \\ 0 & a_1^2+a_2^2 & 0 & a_2a_3 & 0 & \dots & \dots & \dots & \dots & 0 \\ a_1a_2 & 0 & a_2^2+a_3^2 & 0 & a_3a_4 & \dots & \dots & \dots & \dots & 0 \\ 0 & a_2a_3 & 0 & a_3^2+a_4^2 & 0 & \dots & \dots & \dots & \dots & 0 \\ 0 & 0 & a_3a_4 & 0 & a_4^2+a_5^2 & \dots & \dots & \dots & \dots & 0 \\ 0 & 0 & 0 & a_4a_5 & 0 & \dots & \dots & \dots & \dots & 0 \\ 0 & 0 & 0 & 0 & a_5a_6 & \dots & \dots & \dots & \dots & 0 \\ 0 & 0 & 0 & 0 & \dots & \dots & \dots & \dots & \dots & 0 \\ 0 & \dots & \dots & \dots & \dots & \dots & \dots & \dots & \dots & 0 \\ 0 & \dots & \dots & \dots & \dots & \dots & \dots & \dots & \dots & 0 \\ 0 & \dots & \dots & \dots & \dots & \dots & \dots & \dots & \dots & a_{Q-2}a_{Q-1} \\ 0 & \dots & \dots & \dots & \dots & \dots & \dots & \dots & \dots & 0 \\ 0 & 0 & 0 & 0 & 0 & \dots & \dots & \dots & \dots & a_{Q-1}^2 \end{pmatrix} \tag{5}$$

FIG. 3. $P(x)\ vs.\ x$ for $d = 1, R = 10, k = 20\pi$

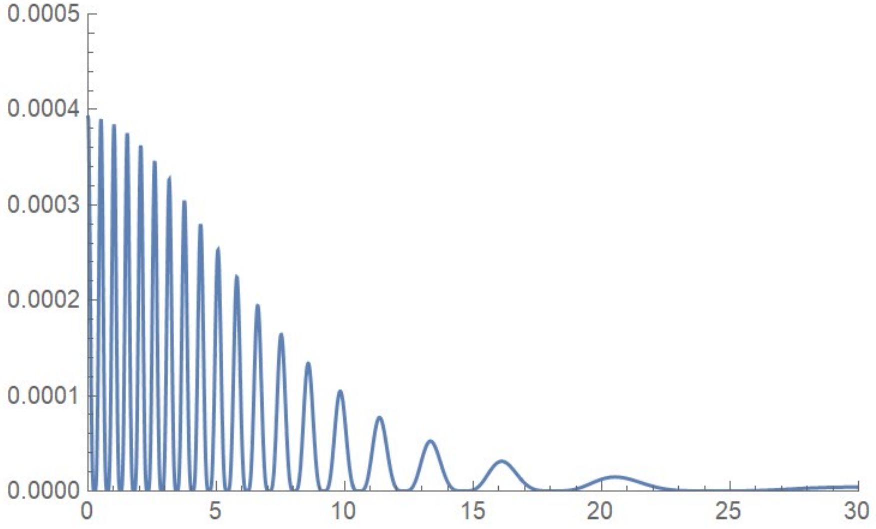

For an arbitrary vector x, with components $x_1, x_2, ..., x_Q$ and the definitions $a_{-1} = a_0 = a_Q = a_{Q+1} = 0$ and $x_{-1} = x_0 = x_{Q+1} = x_{Q+2} = 0$, we write the scalar as

$$\begin{pmatrix} x_1 & x_2 & . & x_Q \end{pmatrix} \begin{pmatrix} a_0^2+a_1^2 & 0 & a_1a_2 & 0 & 0 & \dots\ \dots\ \dots\ \dots & 0 \\ 0 & a_1^2+a_2^2 & 0 & a_2a_3 & 0 & \dots\ \dots\ \dots\ \dots & 0 \\ a_1a_2 & 0 & a_2^2+a_3^2 & 0 & a_3a_4 & \dots\ \dots\ \dots\ \dots & 0 \\ 0 & a_2a_3 & 0 & a_3^2+a_4^2 & 0 & \dots\ \dots\ \dots\ \dots & 0 \\ 0 & 0 & a_3a_4 & 0 & a_4^2+a_5^2 & \dots\ \dots\ \dots\ \dots & 0 \\ 0 & 0 & 0 & a_4a_5 & 0 & \dots\ \dots\ \dots\ \dots & 0 \\ 0 & 0 & 0 & 0 & a_5a_6 & \dots\ \dots\ \dots\ \dots & 0 \\ 0 & 0 & 0 & 0 & \dots & \dots\ \dots\ \dots\ \dots & 0 \\ 0 & \dots & \dots & \dots & \dots & \dots\ \dots\ \dots\ \dots & 0 \\ 0 & \dots & \dots & \dots & \dots & \dots\ \dots\ \dots\ \dots & 0 \\ 0 & \dots & \dots & \dots & \dots & \dots\ \dots\ \dots\ \dots & a_{Q-2}a_{Q-1} \\ 0 & \dots & \dots & \dots & \dots & \dots\ \dots\ \dots\ \dots & 0 \\ 0 & 0 & 0 & 0 & 0 & \dots\ \dots\ \dots\ \dots & a_{Q-1}^2+a_Q^2 \end{pmatrix} \begin{pmatrix} x_1 \\ x_2 \\ . \\ . \\ . \\ . \\ . \\ . \\ . \\ . \\ . \\ . \\ x_Q \end{pmatrix}$$

Hence

$$\begin{aligned} x^T\mathcal{G}x = \Big(& a_{-1}a_0x_{-1}x_1 + (a_0^2 + a_1^2)x_1^2 + a_1a_2x_1x_3 \\ & + a_0a_1x_0x_2 + (a_1^2 + a_2^2)x_2^2 + a_2a_3x_2x_4 \\ & + a_1a_2x_1x_3 + (a_2^2 + a_3^2)x_3^2 + a_3a_4x_3x_5 \\ & + \dots \\ & + a_{Q-2}a_{Q-1}x_{Q-2}x_Q + (a_{Q-1}0^2 + a_Q^2)x_1^2 + a_Qa_{Q+1}x_Qx_{Q+2}\Big) \\ = {} & a_1^2x_2^2 + a_{Q-1}^2x_Q^2 + (a_1x_1 + a_2x_3)^2 + (a_2x_2 + a_3x_4)^2 + \dots + (a_{Q-2}x_{Q-2} + a_{Q-1}x_Q)^2 \geq 0 \end{aligned}$$

and is true $\forall x$. The matrix is, hence, positive-definite.

APPENDIX III

In order to produce a microscopic basis for Equation (3), one should look at an elementary process in a very localized volume near a slit, from the following term in the QED action

$$\mathcal{A}_{int} = -ie \int d^4x\ \bar{\psi}\,\gamma^\mu A^\mu \psi \tag{6}$$

Consider the physics of the situation with the electron. Initially it is delocalized between the slits; post interaction with the photomultiplier's field, it gets localized to the slit 1. The dimensions of the slit is usually compared to the electron's Compton wavelength (see Jonsson *al*).

Hence, considering Equation (3), the process is that electron comes into a region, with energy ω_e, leaves with energy ω_e and also causes the emission of a photon of energy ω_p. Some energy was added in the process. The energy addition must come through the interaction of the electron with a background field. The background field could be considered very concentrated in the region around slit 1. We also note that for the geometries considered here, $\omega_p \ll \omega_e$, as we only need to localize the electron to within a μ or so with photons, while the electron wave's wavelength is much shorter.

To this end, we consider a second-order process, with a very intense (localized in space around the slit 1) auxiliary field $A_\mu^{classical}$ which is large but constant in the region of slit 1 and also constant in time. We then get the approximate second-order term below (as well as its hermitian conjugate), with the usual annihilation/creation operators for photons $(a, a^\dagger)$ and electrons $(c, c^\dagger)$, and time averaged over

$$\begin{aligned} &= -e^2 \Delta V \Delta t \;\; \bar{\psi}(\vec{x}_1) \not{A} \psi(\vec{x}_1) \;\; < \bar{\psi}(\vec{x}_1) \not{A}^{classical} \psi(\vec{x}_1) > \\ &= (constants)\; c^\dagger(\vec{x}_1) e^{-i\omega_e t} \;\; \times \;\; a^\dagger e^{-i\omega_p t} \;\; \times \;\; c(\vec{x}_1) e^{i\omega_e t} \\ &\approx \Gamma_1 e^{-i\omega_p t} a^\dagger c_1^\dagger c_1 \end{aligned} \tag{7}$$

which is what we used (along with its hermitian conjugate) in the rather idealized calculation (Equation 3).

It is clear from the above that energy is being added by the background field.

FIG. 4. Electron Scattering Off an background field and producing a photon - here $\omega_\gamma = \omega_p$

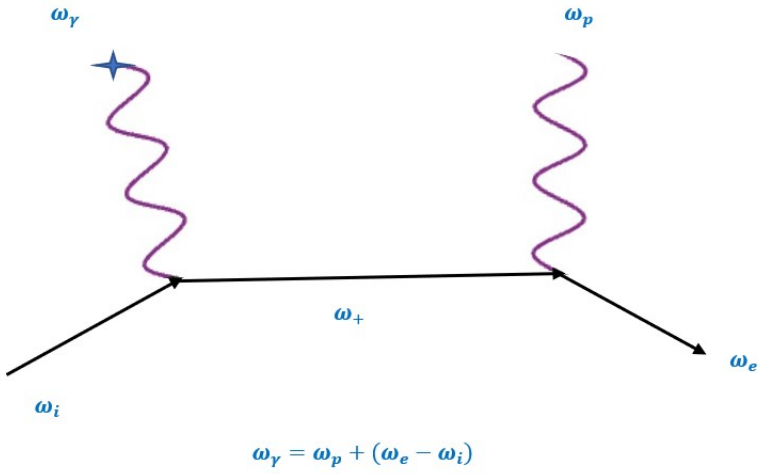

Supplement: Supplementary file 1 — Supplementary Information. [file 41598_2021_99995_MOESM1_ESM.pdf]
